# Supplementary material for: Development and Psychometric Validation of the Breast Cancer Stigma Assessment Scale for Women with Breast Cancer and Its Survivors
Source: Healthcare (Basel). 2024 Feb 6;12(4):420. doi: 10.3390/healthcare12040420 (PMC10887980; doi:10.3390/healthcare12040420)
Supplement: Supplementary file 1 [file healthcare-12-00420-s001.zip › healthcare-2773345-supplementary.pdf]

Anexo Escala:

BCSAS (Breast Cancer Stigma Scale)

Las afirmaciones a continuación describen experiencias relacionadas con el cáncer de mama. Están dirigidas a mujeres en fase activa de la enfermedad y también a supervivientes. Conteste, sin detenerse demasiado, la respuesta que mejor coincide con usted en el momento actual. No hay respuestas correctas o incorrectas. De las afirmaciones propuestas, puntúe de 1 a 5 en función del grado de acuerdo o desacuerdo.

	1	2	3	4	5
1. Oculto o minimizo mi enfermedad con algunas personas.					
2. No me gusta que algunas personas me traten de forma distinta por el cáncer de mama.					
3. Me arrepiento de haber contado a algunas personas que tengo cáncer de mama.					
4. En algunas situaciones me da vergüenza decir que tengo cáncer de mama.					
5. Desde el cáncer de mama, a veces me siento aislada del resto del mundo.					
6. El cáncer de mama me perjudica o limita laboralmente.					
7. Prefiero evitar ciertos lugares desde que tengo cáncer de mama.					
8. Me han molestado algunas actitudes o comportamientos de personas que saben de mi cáncer de mama.					
9. Me siento incómoda con las miradas, el morbo o la curiosidad de algunas personas.					
10. No me gusta que algunas personas sientan lástima por mí.					
11. Siento que no soy tan válida como las demás porque tengo cáncer de mama.					
12. Me esfuerzo por ocultar o disimular los cambios físicos derivados del cáncer de mama.					
13. Cuando tienes cáncer de mama, la caída del cabello o las secuelas físicas son una preocupación importante.					
14. Creo que mi forma de ser o situaciones de mi vida pudieron provocar mi cáncer de mama.					
15. Creo que tener cáncer de mama ha sido un aviso de que era necesario cambiar algunos aspectos de mi vida y de mi misma.					
16. Me resulta desagradable que algunas personas se sientan incómodas o me eviten a causa del cáncer de mama.					
17. Si pienso que tengo cáncer en mi cuerpo siento asco.					
18. No me gusta cuando la gente evita decir o escuchar la palabra cáncer.					
19. En algunos momentos me ha resultado difícil decir y/o escuchar la palabra cáncer.					
20. Con frecuencia siento miedo o preocupación porque me siento en peligro a causa del cáncer.					
21. Siento que no soy la misma que era antes del cáncer de mama.					
22. Tener cáncer ha marcado un antes y un después en mi vida.					
23. Tener cáncer de mama perjudica las relaciones sexuales.					
24. Tener cáncer de mama interfiere en las relaciones familiares.					
25. Tener cáncer de mama afecta negativamente a las relaciones de pareja.					
26. Me preocupa cómo mi enfermedad afecta a las personas que cuidan de mí.					
27. No me gusta o evito participar en grupos o actividades donde tengo que estar con otras personas con cáncer.					
28. Me resulta difícil afrontar que puede haber dificultades o imposibilidad de ser madre en el futuro a causa del cáncer.					

BCSAS (Breast Cancer Stigma Scale): English Translated

The statements below describe experiences related to breast cancer. They are aimed at women in the active phase of the disease and also at survivors. Answer, without stopping too much, the answer that best matches you at the current moment. There are not correct or incorrect answers. For the proposed statements, rate them from 1 to 5 depending on the degree of agreement or disagreement.

	1	2	3	4	5
1. I hide or minimize my disease with some people.					
2. I don't like that some people treat me differently because of breast cancer.					
3. I regret having told some people that I have breast cancer					
4. In some situations I am embarrassed to say that I have breast cancer.					
5. Since breast cancer, I sometimes feel isolated from the rest of the world.					
6. My breast cancer has a negative or limiting effect on me in my work.					
7. I prefer to avoid certain places since I have breast cancer.					
8. I have been bothered by some attitudes or behaviors of people who know about my breast cancer.					
9. I feel uncomfortable with the stares, morbidness, or curiosity of some people.					
10. I don't like that some people feel sorry for me.					
11. I feel that I am not as valid as others because I have breast cancer.					
12. I make an effort to hide or disguise physical changes resulting from breast cancer.					
13. When you have breast cancer, hair loss or physical sequelae are a major concern.					
14. I believe that my way of being or situations in my life could have caused my breast cancer.					
15. I think having breast cancer has been a wake-up call that I needed to change some aspects of my life and myself.					
16. I find it unpleasant that some people feel uncomfortable or avoid me because of breast cancer.					
17. If I think that I have cancer in my body, I feel disgusted.					
18. I don't like it when people avoid saying or hearing the word cancer.					
19. At times I have found it difficult to say and/or hear the word cancer.					
20. I often feel afraid or worried because I feel in danger because of cancer.					
21. I feel I am not the same as I was before breast cancer.					
22. Having cancer has marked a before and after in my life.					
23. Having breast cancer harms sexual relations.					
24. Having breast cancer interferes with family relationships.					
25. Having breast cancer negatively affects relationships with a partner.					
26. I worry about how my disease affects the people who care for me.					
27. I do not like or avoid participating in groups or activities where I have to be with other people with cancer.					
28. I find it hard to face the fact that I may have difficulty or be unable to be a mother in the future because of cancer.					
